# Prevalence and Predictors of Gastrostomy Tube and Tracheostomy Placement in Anoxic/Hypoxic Ischemic Encephalopathic Survivors of In-Hospital Cardiopulmonary Resuscitation in the United States

**DOI:** 10.1371/journal.pone.0132612

**Published:** 2015-07-21

**Authors:** Veerajalandhar Allareddy, Sankeerth Rampa, Romesh P. Nalliah, Natalia I. Martinez-Schlurmann, Karen B. Lidsky, Veerasathpurush Allareddy, Alexandre T. Rotta

**Affiliations:** 1 UH Rainbow Babies & Children’s Hospital, Case Western Reserve University, Cleveland, Ohio, United States of America; 2 University of Nebraska, Health Services and Research department, Omaha, Nebraska, United States of America; 3 University of Michigan, College of Dentistry, Ann Arbor, Michigan, United States of America; 4 University of Iowa, School of Dentistry, College of Dentistry and Dental Clinics, Iowa City, Iowa, United States of America; Zhongshan Hospital Fudan University, CHINA

## Abstract

**Introduction:**

Current prevalence estimates of gastrostomy tube (GT) /tracheostomy placement in hospitalized patients with anoxic/hypoxic ischemic encephalopathic injury (AHIE) post cardiopulmonary resuscitation (CPR) are unknown. We sought, to estimate the prevalence of AHIE in hospitalized patients who had CPR and to identify patient/hospital level factors that predict the performance of GT/tracheostomy in those with AHIE.

**Methods:**

We performed a retrospective analysis of the Nationwide Inpatient Sample (years 2004–2010). All patients who developed AHIE following CPR were included. In this cohort the odds of having GT and tracheostomy was computed by multivariable logistic regression analysis. Patient and hospital level factors were the independent variables.

**Results:**

During the study period, a total of 686,578 CPR events occurred in hospitalized patients. Of these, 94,336 (13.7%) patients developed AHIE. In this AHIE cohort, 6.8% received GT and 8.3% tracheostomy. When compared to the 40–49 yrs age group, those aged >70 yrs were associated with lower odds for GT (OR = 0.65, 95% CI:0.53–0.80, p<0.0001). Those aged <18 years & those >60 years were associated with lower odds for having tracheostomy when compared to the 40–49 years group (p<0.0001). Each one unit increase in co-morbid burden was associated with higher odds for having GT (OR = 1.23,p<0.0001) or tracheostomy (OR = 1.17, p<0.0001). Blacks, Hispanics, Asians/Pacific Islanders, and other races were associated with higher odds for having GT or tracheostomy when compared to whites (p<0.05). Hospitals located in northeastern regions were associated with higher odds for performing GT (OR = 1.48, p<0.0001) or tracheostomy (OR = 1.63, p<0.0001) when compared to those in Western regions. Teaching hospitals (TH) were associated with higher odds for performing tracheostomy when compared to non-TH (OR = 1.36, 1.20–1.54, p<0.0001).

**Conclusions:**

AHIE injury occurs in a significant number of in-hospital arrests requiring CPR. Certain predictors of GT/ Tracheostomy placement are identified. Patients in teaching hospitals were more likely to receive tracheostomy than their counterparts.

## Introduction

In-hospital cardiac arrests (IHCA) needing cardiopulmonary resuscitation (CPR) are common occurrences in hospitalized patients in the United States (U.S) and worldwide. In a large epidemiological study, the rate of in-hospital CPR in adults in the U.S. had gradually increased over the past decade ranging from 1 CPR event per 453 hospitalized patients in the year 2000 to 1 per 339 in the year 2009 [1]. In adults, data from the 433 hospitals participating in the Get With The Guidelines-Resuscitation registry (GWTG-R) revealed that the mean treated IHCA event rate was 0.92/1000 bed days and approximately 200,000 treated cardiac arrests occur annually in hospitalized patients in the U.S [2]. Although, the survival to discharge rates for adults with treated IHCA were low at less than 20% in several studies [2–5], a recent study from the GWTG-R revealed that the risk adjusted rates of survival to discharge had actually increased from 13.7% in 2000 to 22.3% in 2009 [6]. Although, the rates of clinically significant neurologic disability among adult survivors of treated IHCA decreased over time, with a risk-adjusted rate of 32.9% in 2000 to 28.1% in 2009, it is still considerable due to the high morbidity associated with neurological deficit [6,7,8].

The incidence of IHCA in children varies based on the location, ranging from 0.7% to 3% of all pediatric hospital admissions to 1.8% to 5.5% of pediatric intensive care unit admissions [9–13]. In children, over the past decade the risk adjusted rates of survival to discharge of treated IHCA increased from 14.3% in 2000 to 43.4% in 2009, and importantly, similar to the adults, the survival trends were not accompanied by higher rates of neurological disability among survivors over time [14].

The clinical manifestations of the brain injury range from minimal memory deficits to persistent vegetative state or even brain death. Severe brain injury in survivors of IHCA post CPR is a major cause of morbidity [12]. Gastrostomy tube &/or tracheostomy placement may be needed in certain cohorts of AHIE patients who are not expected to make rapid clinical progress at least during their present hospitalization. Recipients of technology dependent, chronic life sustaining processes, especially those needing prolonged mechanical ventilation, are a unique population group whose medical needs and resource utilization are considerable [15–17]. Current large population based prevalence estimates and predictors of gastrostomy tube (GT) /tracheostomy placement in hospitalized patients with anoxic/hypoxic ischemic encephalopathic injury (AHIE) post cardiopulmonary resuscitation (CPR) in any age group are unclear. It is imperative to know such estimates so as to optimize health care utilization.

The objectives of the present study are two-fold. To estimate the prevalence of anoxic/hypoxic ischemic encephalopathy in survivors of in-hospital cardiac arrests of any cause post cardiopulmonary resuscitation. Further, we sought to identify patient and hospital levels factors that predict the placement of gastrostomy tube and/or tracheostomy in those with AHIE. We hypothesize that a mix of patient/hospital level factors could predict the performance of these procedures in this cohort.

## Materials and Methods

### Database, Design, Institutional Review Board and Data User Agreement

We performed a retrospective analysis of the Nationwide Inpatient Sample (NIS) for the years 2004 to 2010 [18]. The NIS is the largest publicly available all-payer inpatient health care database in the United States, yielding national estimates of hospital inpatient stays. The NIS is a component database of the Healthcare Cost and Utilization Project (HCUP) sponsored by the Agency for Healthcare Research and Quality (AHRQ) [18]. Unweighted, it contains data from more than 7 million hospital stays each year. Weighted, it estimates more than 36 million hospitalizations nationally. The NIS is drawn from 44 States participating in the Health care utilization project (HCUP), representing more than 95% of the U.S. population. The NIS approximates a 20-percent stratified sample of discharges from U.S. community hospitals, excluding rehabilitation and long-term acute care hospitals. The NIS contains several clinical and nonclinical data elements for each hospital stay, including, primary and secondary diagnoses and procedures, patient demographic characteristics (e.g., sex, age, race, median household income etc), hospital characteristics (e.g., ownership), expected payment source, total charges, discharge status and disposition, length of stay, severity and comorbidity measures [18]. This study was granted ‘exempt’ status by the Institutional Review Board of The University of Iowa. Research studies involving the collection or study of existing data, documents, records, pathological specimens, or diagnostic specimens, if these sources are publicly available or if the information is recorded by the investigator in such a manner that subjects cannot be identified, directly or through identifiers linked to the subjects are permitted to be classified as research that is “exempt” from IRB full or expedited review. The present study was a retrospective analysis of hospital based de-identified administrative discharge dataset that is available publicly for purchase from the AHRQ. The first author (VJA) completed the data user agreement with HCUP-AHRQ and obtained the pertinent datasets. As per the data user agreement, cell counts ≤10 cannot be reported to maintain patient privacy. In accordance with the agreement, low cell counts are not reported and the term “DS” (Discharge information suppressed) is used instead, where appropriate.

### Patient Selection

All hospitalized patients who had an in-hospital cardiac arrest of any cause and received cardiopulmonary resuscitation (CPR) were identified. The ICD-9-CM procedure codes used for identifying patients who had CPR were 99.60 (Cardiopulmonary resuscitation, not specified) and 99.63 (Closed chest cardiac massage). The use of ICD 9 codes has been well described in prior publications [1,19]. In this cohort, those who developed anoxic/hypoxic ischemic encephalopathy (AHIE) were selected using ICD-9-CM diagnosis codes of 348.1 (Anoxic brain damage) [20]. This group who had anoxic/hypoxic ischemic encephalopathy and had underwent a CPR comprised the final study population for the present study.

### Outcome Variables

Primary outcomes included placement of gastrostomy tube or tracheostomy in the AHIE cohort. Performance of Gastrostomy tube placement procedure was identified by using the ICD-9-CM procedure codes: 43.11 (percutaneous endoscopic gastrostomy/ percutaneous transabdominal gastrostomy) or 43.19 (other gastrostomy). Performance of tracheostomy was identified by using the ICD-9-CM procedure codes: 31.1 (temporary tracheostomy), 31.2 (permanent tracheostomy), 31.21 (mediastinal tracheostomy), or 31.29 (other permanent tracheostomy) [20].

### Statistical Analysis

Multivariable logistic regression analysis was used to examine the association between placement of gastrostomy tube and tracheostomy and multiple patient and hospital level factors.

Separate multivariable models were used for gastrostomy and tracheostomy procedures. In both these models, the effects of clustering of outcomes were adjusted. Age, sex, race, co-morbid burden, hospital region, and teaching status were the independent variables of interest in the regression models. A sensitivity analysis was also conducted wherein the insurance status (Medicare, Medicaid, Private Insurance, Other insurance and the uninsured) was also included in the multivariable regression analysis. Co-morbid burden was computed by summing the presence of 29 different chronic co-morbid conditions in this cohort of patients. The co-morbid conditions examined included: AIDS, alcohol abuse, deficiency anemias, rheumatoid arthritis/collagen vascular diseases, chronic blood loss anemia, congestive heart failure, chronic pulmonary disease, coagulopathy, depression, diabetes—uncomplicated, diabetes—with chronic complications, drug abuse, hypertension, hypothyroidism, liver disease, lymphoma, fluid and electrolyte disorders, metastatic cancer, neurological disorders, obesity, paralysis, peripheral vascular disorders, psychoses, pulmonary circulatory disorders, renal failure, solid tumor without metastasis, peptic ulcer disease, valvular disease and weight loss. All tests of associations were two sided and a p-value of <0.05 was deemed to be statistically significant. Statistical tests were conducted using SAS Version 9.3 software (SAS Institute, Cary, NC, USA) and SAS Callable SUDAAN 10.0.1 software (Research Triangle Institute, NC, US).

## Results

**Fig 1:**
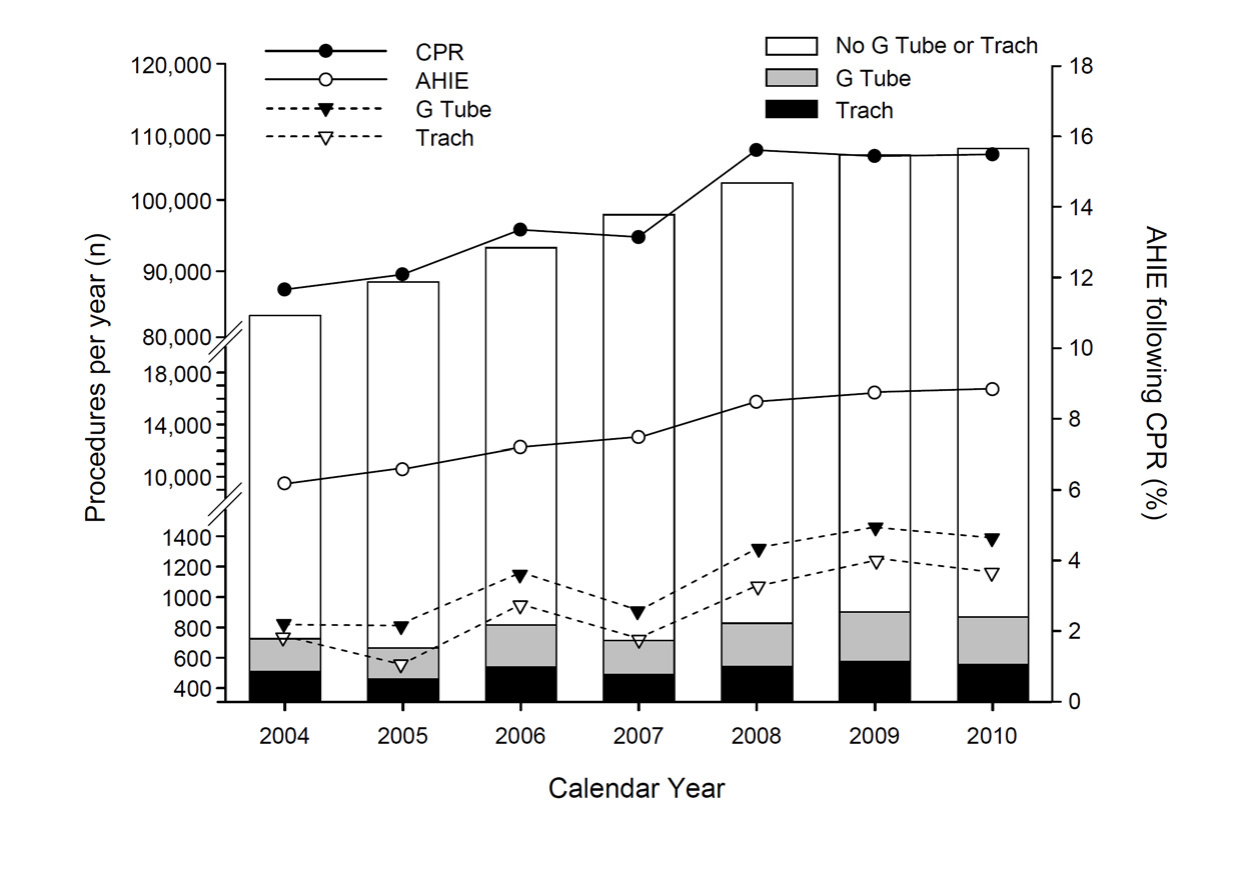
The left axis depicts the number of gastrostomy tube (G Tube, black triangles) and tracheostomy (Trach, white triangles) procedures relative to the number of cardiopulmonary resuscitation (CPR, black circles) events and acute hypoxic ischemic encephalopathy (AHIE, white circles) between the years of 2004 and 2010 (inclusive). The right axis depicts the percentage of patients who developed AHIE following CPR and the proportion of those receiving a G Tube, Trach, or no G Tube or Trach.

During the study period, a total of 686,578 hospitalizations had a CPR event. Of these, 13.7% (N = 94,336) had AHIE. In the cohort of patients with AHIE, 6.8% had gastrostomy tube placement and 8.3% had tracheostomy procedures during the hospitalization. The characteristics of this cohort of patients who had a CPR procedure and AHIE are summarized in Table 1. Those aged 70 years and above comprised 40.1% and those aged 60 to 69% comprised 21.8% of all patients. Only 2% of all patients were aged <18 years. Males comprised the majority of patients (55%). The predominating race was Whites (60.5%), followed by Blacks (21.6%) and Hispanics (10.5%). Medicare was the primary payer for majority of patients (57.1%). A vast proportion of hospitalizations occurred on an emergency/urgent basis (92.1%). Close to 95% of patients had at least one co-morbid condition. Teaching hospitals treated 56.3% of patients. Hospitals located in the southern regions accounted for 38.4% of all hospitalizations. Disposition status of the AHIE cohort included: 77.9% died in the hospital and 14% were discharged to a long term care facility. Fig 1 depicts the trends in the number of gastrostomy tube and tracheostomy procedures relative to the number of cardiopulmonary resuscitation events and acute hypoxic ischemic encephalopathy between the years of 2004 and 2010. The right axis in the Fig 1 depicts the percentage of patients who developed AHIE following CPR and the proportion of those receiving a G Tube, Tracheostomy, or no G Tube or Tracheostomy. Overall, there was a gradual yearly increase in the number of AHIE events post CPR with accompanying steady increments in G tube/Tracheostomy placements.

**Table 1:** Characteristics of Patients with AHIE and who had a CPR.

Characteristics		Total N = 94,336
Had Tracheostomy	Yes	8.3%
Had Gastrostomy	Yes	6.8%
Age	<18 Years	2%
	19 to 29 Years	3.2%
	30 to 39 Years	4.8%
	40 to 49 Years	9.7%
	50 to 59 Years	18.4%
	60 to 69 Years	21.8%
	> 70 Years	40.1%
Sex	Male	55%
	Female	45%
Race (information on race was available for 79,294 patients)	White	60.5%
	Black	21.6%
	Hispanic	10.5%
	Asian/Pacific Islander	3.8%
	Native American	0.6%
	Other Races	2.9%
Insurance Status	Medicare	57.1%
	Medicaid	13.2%
	Private	20.3%
	Uninsured	6.5%
	Other Insurance	2.8%
Disposition Status	Routine discharge	2.8%
	Discharged to another short term hospital	3.1%
	Discharged to a long term care facility (eg: Skilled nursing home)	14%
	Discharged to home health care	1.8%
	Discharged against medical advice	0.1%
	Died in hospital	77.9%
	Unknown destination	0.2%
Type of Admission	Emergency/Urgent	92.1%
	Elective	7.9%
Co-morbid burden	0	4.9%
	1	13.3%
	2	18.6%
	3	19.7%
	4	16.2%
	5	11.8%
	> 6	15.5%
Teaching Status of Hospital	Non-teaching	56.3%
	Teaching	43.7%
Region of Hospital	Northeast	16.8%
	Midwest	19.3%
	South	38.4%
	West	25.5%

The results of the multivariable logistic regression analysis examining the simultaneous association between patient/hospital level characteristics and odds of having a gastrostomy procedure performed during the hospitalization are summarized in Table 2. Those aged 70 years and above were associated with a lower odds for having gastrostomy when compared to those aged 40 to 49 years (OR = 0.65, 95% CI = 0.53–0.80, p<0.0001). Blacks (OR = 2.00, 95% CI = 1.72–2.34, p<0.0001), Hispanics (OR = 1.48, 95% CI = 1.19–1.84, p<0.0001), Asian/Pacific Islanders (OR = 1.67, 95% CI = 1.25–2.22, p<0.0001), and other races (OR = 1.89, 95% CI = 1.37–2.62, p<0.0001) were associated with higher odds for having gastrostomy when compared to Whites. Each one unit increase in co-morbid burden was associated with increasing odds for having gastrostomy procedure (OR = 1.23, 95% CI = 1.01–1.50, p<0.0001). Hospitals located in the northeastern (OR = 1.48, 95% CI = 1.21–1.81, p<0.0001) were associated with higher odds for doing gastrostomy while hospitals located in the southern regions were associated with lower odds for doing gastrostomy (OR = 0.75, 95% CI = 0.63–0.90, p<0.0001) when compared to hospitals in the western region. Patients who had Medicare (OR = 1.48, 95% CI {1.05–2.09}, p = 0.03), Medicaid (OR = 2.04, 95% CI {1.43–2.89}, p<0.0001), or private insurance (OR = 1.50, 95% CI {1.07–2.10}, p = 0.02), were more likely to receive gastrostomy tube compared to the uninsured (reference).

**Table 2:** Multivariable Logistic Regression for Performance of Gastrostomy.

Variables		Multivariable Model without Insurance Status		Multivariable Model with Insurance Status	
		Odds Ratio (95% CI)	p-value	Odds Ratio (95% CI)	p-value
Age	<18 Years	1.38 (0.90–2.10)	0.14	1.25 (0.82–1.91)	0.30
	19 to 29 Years	1.08 (0.76–1.54)	0.65	1.05 (0.74–1.48)	0.80
	30 to 39 Years	0.83 (0.62–1.13)	0.23	0.85 (0.62–1.14)	0.28
	40 to 49 Years	Reference Group		Reference Group	
	50 to 59 Years	0.94 (0.76–1.16)	0.56	0.94 (0.76–1.17)	0.58
	60 to 69 Years	0.84 (0.69–1.04)	0.11	0.87 (0.70–1.07)	0.18
	> 70 Years	0.65 (0.53–0.80)	<0.0001	0.69 (0.55–0.86)	<0.0001
Sex	Female	0.98 (0.87–1.10)	0.72	0.97 (0.86–1.08)	0.56
	Male	Reference Group		Reference Group	
Race	Black	2.00 (1.72–2.34)	<0.0001	1.95 (1.67–2.28)	<0.0001
	Hispanic	1.48 (1.19–1.84)	<0.0001	1.44 (1.16–1.79)	<0.0001
	Asian/Pacific Islander	1.67 (1.25–2.22)	<0.0001	1.62 (1.21–2.17)	<0.0001
	Native American	1.11 (0.42–2.94)	0.83	1.09 (0.41–2.89)	0.87
	Other Races	1.89 (1.37–2.62)	<0.0001	1.85 (1.33–2.56)	<0.0001
	White	Reference Group		Reference Group	
Co-morbid burden	1 unit increase	1.23 (1.01–1.50)	<0.0001	1.18 (1.15–1.22)	<0.0001
Hospital Region	Northeast	1.48 (1.21–1.81)	<0.0001	1.49 (1.22–1.82)	<0.0001
	Midwest	0.88 (0.72–1.08)	0.23	0.89 (0.73–1.09)	0.26
	South	0.75 (0.63–0.90)	<0.0001	0.78 (0.65–0.93)	<0.00001
	West	Reference Group		Reference Group	
Teaching Status	Teaching Hospital	1.01 (0.88–1.15)	0.93	1.00 (0.88–1.15)	0.94
	Non-teaching Hospital	Reference Group		Reference Group	
Insurance Status	Medicare	-		1.48 (1.05–2.09)	0.03
	Medicaid	-		2.04 (1.43–2.89)	<0.0001
	Private insurance	-		1.50 (1.07–2.10)	0.02
	Other insurance	-		1.51 (0.95–2.39)	0.08
	Uninsured	-		Reference Group	

The results of the multivariable logistic regression analysis examining the simultaneous association between patient/hospital level characteristics and odds of having a tracheostomy procedure performed during the hospitalization are summarized in Table 3. Those aged 70 years and above (OR = 0.53, 95% CI = 0.45–0.63, p<0.0001), aged 60 to 69 years (OR = 0.74, 95% CI = 0.62–0.89, p<0.0001), and those aged <18 years (OR = 0.43, 95% CI = 0.27–0.68, p<0.0001) were associated with lower odds for having tracheostomy when compared to those aged 40 to 49 years. Blacks (OR = 2.05, 95% CI = 1.77–2.37, p<0.0001), Hispanics (OR = 1.72, 95% CI = 1.43–2.07, p<0.0001), Asian/Pacific Islanders (OR = 1.91, 95% CI = 1.45–2.50, p<0.0001), and other races (OR = 1.68, 95% CI = 1.21–2.34, p<0.0001) were associated with higher odds for having tracheostomy when compared to Whites. Each one unit increase in co-morbid burden was associated with increasing odds for having tracheostomy procedure (OR = 1.17, 95% CI = 1.13–1.20, p<0.0001). Hospitals located in the northeastern (OR = 1.63, 95% CI = 1.37–1.95, p<0.0001) were associated with higher odds for doing tracheostomy while hospitals located in the southern regions were associated with lower odds for doing tracheostomy (OR = 0.85, 95% CI = 0.72–0.99, p = 0.04) when compared to hospitals in the western region. Teaching hospitals were associated with a higher odds of doing tracheostomy (OR = 1.36, 95% CI = 1.20–1.54, p<0.0001) when compared to non-teaching hospitals. Patients who had Medicare (OR = 1.37, 95% CI {1.01–1.84}, p = 0.04), Medicaid (OR = 1.70, 95% CI {1.27–2.28}, p<0.0001), or private insurance (OR = 1.44, 95% CI {1.08–1.92}, p = 0.01), were more likely to receive tracheostomy compared to the uninsured (reference).

**Table 3:** Multivariable Logistic Regression for Performance of Tracheostomy.

Variables		Multivariable Model without Insurance Status		Multivariable Model with Insurance Status	
		Odds Ratio (95% CI)	p-value	Odds Ratio (95% CI)	p-value
Age	<18 Years	0.43 (0.27–0.68)	<0.0001	0.40 (0.25–0.64)	<0.0001
	19 to 29 Years	1.03 (0.76–1.38)	0.87	1.00 (0.75–1.35)	0.98
	30 to 39 Years	0.94 (0.72–1.22)	0.64	0.94 (0.72–1.22)	0.64
	40 to 49 Years	Reference Group		Reference Group	
	50 to 59 Years	0.91 (0.76–1.10)	0.33	0.91 (0.76–1.10)	0.34
	60 to 69 Years	0.74 (0.62–0.89)	<0.0001	0.75 (0.62–0.91)	<0.0001
	> 70 Years	0.53 (0.45–0.63)	<0.0001	0.55 (0.45–0.68)	<0.0001
Sex	Female	1.05 (0.95–1.17)	0.36	1.04 (0.93–1.16)	0.47
	Male	Reference Group		Reference Group	
Race	Black	2.05 (1.77–2.37)	<0.0001	2.02 (1.75–2.34)	<0.0001
	Hispanic	1.72 (1.43–2.07)	<0.0001	1.70 (1.41–2.05)	<0.0001
	Asian/Pacific Islander	1.91 (1.45–2.50)	<0.0001	1.88 (1.43–2.47)	<0.0001
	Native American	1.79 (0.83–3.82)	0.13	1.76 (0.82–3.78)	0.15
	Other Races	1.68 (1.21–2.34)	<0.0001	1.65 (1.18–2.31)	<0.0001
	White	Reference Group		Reference Group	
Co-morbid burden	1 unit increase	1.17 (1.13–1.20)	<0.0001	1.16 (1.13–1.20)	<0.0001
Hospital Region	Northeast	1.63 (1.37–1.95)	<0.0001	1.64 (1.37–1.97)	<0.0001
	Midwest	0.93 (0.77–1.13)	0.47	0.94 (0.78–1.14)	0.52
	South	0.85 (0.72–0.99)	0.04	0.87 (0.74–1.01)	0.07
	West	Reference Group		Reference Group	
Teaching Status	Teaching Hospital	1.36 (1.20–1.54)	<0.0001	1.35 (1.20–1.53)	<0.0001
	Non-teaching Hospital	Reference Group		Reference Group	
Insurance Status	Medicare	-		1.37 (1.01–1.84)	0.04
	Medicaid	-		1.70 (1.27–2.28)	<0.0001
	Private insurance	-		1.44 (1.08–1.92)	0.01
	Other insurance	-		1.46 (0.98–2.18)	0.06
	Uninsured	-		Reference Group	

## Discussion

Advances in ICU care and widespread adoption of CPR guidelines have led to increasing survival rates in patients experiencing in-hospital arrests needing CPR [1,2,4]. However, survivors of in-hospital arrests post CPR are an important cohort of population who are at risk of needing chronic critical care. Chronic critical illness (CCI) is now a well-recognized major cause of morbidity, mortality and hospital resource utilization [7,16,17]. Anoxic/hypoxic ischemic encephalopathic survivors requiring technology dependence such as tracheostomy placement (with or without associated ventilator assistance) and/or gastrostomy tube placement are associated with considerable in-hospital resource utilization and frequent post-acute care [16,17]. Using a large population based sample, we show that AHIE occurs in a considerable number of survivors of in-hospital arrests receiving CPR. Although, the yearly rate of AHIE in CPR survivors increased steadily during the study period ranging from a low of 10.1% in 2004 to a high of 15.6% in 2010, G tube/tracheostomy procedural volume remained steady in this AHIE cohort.

In the past decade, in the in-hospital setting, quality measures of CPR effectiveness have evolved from survival rate of individual CPR events, to survival to hospital discharge, to assessment of neurological disability at the time of hospital discharge [6, 21–24]. Girotra et al, identified all adults (>18 years) who had treated IHCA at 374 hospitals that participated in the Get with the guidelines-Resuscitation registry in the past decade and showed that both survival and neurologic outcomes improved. The rates of clinically significant neurologic disability decreased from 32.9% in 2000 to 28.1% in 2009 (adjusted rate ratio per year, 0.98; P = 0.02 trend) [6]. In their study clinical performance category scores (CPC) were used to identify any neurological deficit. A CPC score of 1 included mild or no neurologic disability and CPC score of 5 reflected brain death. In comparison, in the present study, the overall AHIE rate was 13.7% and this relatively lower rate could be due to selection of patients with worse neurological disability since our selection criteria was specifically anoxic/hypoxic ischemic encephalopathy and not any neurological deficits.

In our study we included both adults and children (< 18 years). In the present study, children accounted for only 2% of the AHIE cohort whereas adults > 70 years accounted for the majority of the patients with AHIE. Prior large studies had delineated CPR outcomes for children and adults separately since the cause of arrests needing CPR differs significantly in the age groups [1, 5, 6, 9–12, 14, 19]. Primary cardiac conditions are more frequent cause of arrest needing CPR in adults, whereas respiratory failure leading to arrest predominates in children [12, 14]. In a large multicenter registry of in-hospital cardiac arrests in children and adults, the rate of survival to hospital discharge following pulseless cardiac arrest was higher in children compared to adults (27% vs 18%; [OR]: 2.29), and of these survivors, 65% of children and 73% of adults had good neurological outcome [13]. We chose to include patients of all age range so as to compare and quantify the performance of technology assisted care (specifically, G tube and tracheostomy placement) in neurologically impaired cohort.

The findings from this present study raise further important questions. What is the right rate of G tube/tracheostomy placement in survivors of treated IHCA with AHIE? Or, more importantly, who are the patients who would benefit from such interventions? In ideal circumstances, AHIE patients who are expected to make “meaningful recovery” should receive tracheostomy, G tube or other assisted medical technology and care so that recovery is facilitated or completed. However, this presents two dilemmas- objectively defining meaningful recovery and prognosticating which patients would make such recovery. Prediction of outcomes in comatose survivors after cardiac arrests requiring CPR has been described in several studies [25–28]. Results from these studies are varied, yet important, and much further research is needed before definitive recommendations guide clinical care. Decision for placement of G tube/tracheostomy in AHIE cohort is often complex and depends on several factors, including but not limited to, patient or surrogate preferences (advance directives, parental decisions for children), functional progress (or lack of it) of survivors as perceived by the medical team, co morbidity, and/or family/religious beliefs which precludes withdrawal of care. In the present study, which represents practices beyond single centers, the overall tracheostomy rate was 8.3% and G tube placement rate was 6.7% in the AHIE cohort. Lack of similar large scale studies precludes us from comparing and inferring from the rate identified in our study.

Although, a definite answer to who would benefit from G tube/tracheostomy is beyond the scope of this study, we attempt to fill the knowledge gap to an extent by identifying the predictors of G tube/tracheostomy placement in AHIE cohorts of treated IHCA’s. In our study, patients in extremes of age groups had lower rates for G tube/ tracheostomy placement. Children were less likely to receive tracheostomy compared to adults. Decision making pertaining to placement of G tube/ tracheostomy and/or technological assistance in neurologically impaired children is very complex and is driven by several factors including but not limited to surrogate decision makers preferences (parents, legal guardians), ethics/palliative care and other specialists recommendations, sometimes court orders, and the medical team’s ability to assess and prognosticate neurological recovery. Factors influencing neurological recovery after primary insult to developing brain are incompletely understood at this time and standardized tools to prognosticate recovery in children are not currently available to guide clinical practice. In the present study, older patients (>70 years) were also less likely to receive G tube or tracheostomy. Although, functional recovery leading to lower rates in this age group is a possibility, it is more plausible that older patient’s had worse neurological disability after arrest and the anticipated poor quality of life precluded meaningful technology assistance. The nature of the dataset precludes us from further evaluating the cause(s) of this finding.

Prior studies have shown that African Americans and Hispanics prefer more aggressive life-sustaining procedures and care with associated higher costs compared to Whites [29–31]. In the present study, we identify race as an independent predictor of both G tube and tracheostomy placement in the neurologically disabled cohort post treated IHCA. African Americans, Hispanics, Asian/Pacific Islanders and other races (except Native Americans) were all at higher odds of receiving G tube and tracheostomy compared to Whites. To our knowledge, this is the first study to show racial differences in performance of G tube/tracheostomy in AHIE cohort. The reasons could be patient, family or provider preferences. A prior study has shown that physician’s preferences for end-of-life treatment for themselves in persistent vegetative state or organic brain disease do follow the same pattern by race as patient’s preferences [32]. The reasoning behind the finding of racial differences in G tube/tracheostomy placement in the AHIE cohort is complex and probably multifactorial- including, but not limited to, age, level of education, socioeconomic status, lack of living will or advance directives, recent health status including co-morbidities, perceptions of health-care access, quality of life perceptions, the effectiveness of technology dependent care, cultural beliefs, social networks and the physicians attitudes/experiences of caring for such patients [33–35]. Nevertheless, the consequences are not insignificant since the resource utilization associated with such chronic critical care is considerable [17]. Further research is needed to identify the factors associated with the deeper social reasoning behind the more aggressive treatment preferred by the African Americans, Hispanics and Asian/Pacific Islanders compared to the Caucasian population.

To our knowledge, this is the first study at a large population level to show a significant association between insurance status and performance of gastrostomy tube/tracheostomy in the neurologically impaired population post treated in-hospital cardiac arrest. In the present study, patients who had some form of insurance (Medicaid, Medicare or private) were more likely to receive GT or Tracheostomy compared to the uninsured population. The reasoning behind this finding is again complex and likely multifactorial—high costs associated with lack of insurance in care of such patients and perceptions of health-care access for uninsured. Prior research has shown that critically ill patients in the United States who do not have health insurance receive fewer critical care services including fewer procedures (including tracheostomy {OR, 0.43; 95% CI, 0.29–0.64}), and may experience worse clinical outcomes compared to their counterparts [36,37]. Further studies are warranted to explore the rationale behind this complex association.

Not surprisingly, in our study higher co morbid burden was associated with higher need for both G tube and tracheostomy placement. It is very likely that patients with co morbidity are at a higher risk of de-conditioning post-acute care illness and need technology based assistance such as frequent pulmonary toileting, ventilator support, and delivery of medications/nutrition via G tube to optimize recovery. Further, patients in teaching hospitals were more likely to receive tracheostomy compared to their counterparts. It is plausible that teaching and large hospitals serve as referral centers and care for patients with higher complexity and co morbidity which increased their necessity of performing tracheostomy [38,39]. A prior large population based study had revealed that regional differences exist in care of surgical patients [40]. In our study, a regional variance in performance of G tube/tracheostomy was observed, a finding which merits further investigation.

The strengths of our study pertain to the size and the breath of the sample. The NIS (nationally representative data) reflects practices that are beyond single center experiences and is generalizable. To our knowledge, this is the first study at a population level to identify certain predictors of G tube/tracheostomy placement in neurologically impaired population. In view of raising health care costs associated with CCI [8,16,17] and the increasing rates of discharges to long term care facilities &/or hospice care centers [1,8], it is imperative that patients, providers, policy makers and the general public are kept updated of the current estimates of technology assisted population with neurological disability, so as to optimize outcomes.

Our study has limitations pertaining to the retrospective design and the use of large administrative hospital discharge databases with its potential for billing and coding errors; nevertheless, the NIS is well validated and has been extensively used for population based clinical studies [1,18]. Although, we showed racial differences in placement of G tube/tracheostomy in AHIE cohorts, such findings should be interpreted in the light of the available data pertaining to race in the NIS. Documentation of race is not mandatory in the NIS dataset. In the present study, NIS sample had information on race for only 84% of the AHIE cohort (79,294 out of 94,336 patients who developed AHIE). Nevertheless, several prior studies had demonstrated the use of NIS for showing racial differences in care and outcomes of both surgical and medical conditions [41–43]. The NIS lacks patient specific physiologic, biologic variables and other data that would allow calculation of cerebral performance category scores which are used to evaluate neurologic function of CPR recipients [25,44]. Pertinent clinical information, hospital practices or trach-ventilator rehabilitation bed situation that sometimes may influence the timing of the procedures are unavailable in the NIS dataset, and hence are unaccounted for in this study. Goldberger et al, showed that duration of resuscitation varied between hospitals and suggested that efforts to increase the duration of resuscitation could improve survival in the high risk population [3]. Likewise, a large multicenter registry of IHCA found that the first documented pulseless arrest rhythm was usually asystole or PEA in both adults and children; and because of better survival after asystole and PEA, children had better outcomes than adults despite fewer cardiac arrests due to ventricular tachycardia or ventricular fibrillation [13]. The NIS does not have information pertaining to the duration of CPR and in addition we cannot reliably account for the cause of the arrest. Hence, we are unable to examine these variables.

In addition, post discharge outcomes are not captured by the NIS dataset and hence functional recovery post discharge such as decannulization (removal of a tracheostomy tube) and G tube removal were not assessed in this study. Likewise, post discharge complications or deaths in this cohort were not assessed in this study. Future studies are needed to assess the long term outcomes and quality of life of AHIE recipients of G tube/tracheostomy.

## Conclusion

AHIE injury occurs in a significant number of in-hospital arrests requiring CPR. Certain predictors of G tube/Tracheostomy placement in AHIE cohort are identified. Patients in teaching hospitals were more likely to receive tracheostomy than their counterparts.
